# Effects of Various Drying Techniques on the Quality Attributes and Metabolite Profiles of *Flammulina velutipes* (Fruiting Body) Based on Non-Target Metabolomics

**DOI:** 10.3390/foods15071208

**Published:** 2026-04-02

**Authors:** Wenchan Yang, Yue Su, Huinan Zhou, Lujuan Wang, Danhua Chen, Fengyun Zhao, Jianmin Yun, Xuerui Wang

**Affiliations:** College of Food Science and Engineering, Gansu Agricultural University, Lanzhou 730070, China; 13309335289@163.com (W.Y.); sy20240522@163.com (Y.S.); zhn1975790855@163.com (H.Z.); 18730059795@163.com (L.W.); w15620183970@163.com (D.C.); fyzhao@gsau.edu.cn (F.Z.); yunjianmin@gsau.edu.cn (J.Y.)

**Keywords:** *Flammulina velutipes*, drying method, color, antioxidant capacity, metabolomics

## Abstract

*Flammulina velutipes* (Golden Needle Mushroom, *F. velutipes*) undergoes rapid postharvest deterioration characterized by browning and decay. Drying effectively extends its shelf life and processing window. This study systematically compared the quality attributes and metabolic profiles of *F. velutipes* subjected to different treatments: fresh *F. velutipes* as the control group (CK), hot-air drying (HAD), vacuum freeze drying (VFD), and natural air drying (NAD), to elucidate the underlying mechanisms of drying-induced changes. In appearance, VFD samples were uniformly bright with shape well maintained, while HAD and NAD were brownish yellow with significantly reduced volume. In terms of antioxidant capacity, VFD demonstrated the highest level, followed by HAD. A total of 2645 metabolites were identified in dried *F. velutipes* via metabolomics (positive/negative ion modes), primarily comprising lipids, terpenoids, polyphenols, amino acids, carbohydrates, and steroids. In contrast to VFD, both HAD and NAD showed reduced levels of certain metabolites. VFD treatment yielded the richest profile of differential metabolites in *F. velutipes*. These results position VFD as the superior method for preserving the quality and metabolic integrity in *F. velutipes*. This comparative study serves as a practical reference for selecting the most suitable drying method in the *F. velutipes* industry and enhances our understanding of the metabolic responses to dehydration stress.

## 1. Introduction

China is the global leading producer of *Flammulina velutipes* (*F. velutipes*) [1]. The widespread popularity of *F. velutipes* is largely attributed to its remarkable nutritional value, as it is rich in a variety of bioactive compounds, including polysaccharides, essential amino acids, polyphenols, and glycoproteins [2,3,4,5]. These compounds contribute to various health benefits, including anti-inflammatory, antioxidant, hypolipidemic, and anti-tumor properties [6,7,8], as well as immune modulation through cytokine induction [9]. However, the high moisture content (up to 90%) of fresh *F. velutipes* makes it highly perishable, leading to post-harvest browning, decay, and consequent economic and nutritional losses. To mitigate this issue, drying is widely employed industrially to reduce moisture to safe levels for storage.

Common drying techniques for *F. velutipes* include natural air drying (NAD), hot air drying (HAD), and vacuum freeze drying (VFD), which represent traditional, thermal, and low-temperature dehydration methods widely used in mushroom processing. These techniques are commonly applied in the food industry and provide distinct drying mechanisms, making them suitable for comparative studies on quality changes during dehydration. HAD is cost-effective, easy to operate and suitable for large-scale processing; however, it is often time-consuming and may lead to quality degradation [10]. Conversely, VFD excellently preserves nutrients, structure, and rehydration capacity; its high energy consumption and low efficiency limit widespread application [11]. NAD, while simple and low-cost, lacks control over environmental factors, posing risks of contaminants and inconsistent quality [12]. In recent years, research on the drying of edible fungi has primarily focused on how different drying methods influence their nutritional composition, sensory attributes, antioxidant activity, and flavor profile. For instance, Yang et al. [13] employed HS-SPME/GC-MS to investigate the impact of various HAD processes on the flavor compounds (TFC) and Maillard reaction products of boletus. Similarly, Meng et al. [14] examined the effects of four drying techniques on both non-volatile nutrients and volatile aroma compounds in *Gomphus luteolus*. These studies indicate that the drying technique critically affects product quality. Nevertheless, the mechanisms underlying these quality changes, especially at the metabolic pathway level, remain poorly understood. Understanding these mechanisms is essential for selecting appropriate drying processes and improving the nutritional and sensory quality of dried *F. velutipes*.

Metabolomics has emerged as a powerful tool for comprehensively identifying and quantifying metabolites, enabling the systematic comparison of metabolite profiles and elucidation of underlying biological mechanisms. Metabolomics has been extensively applied in plant and food science. For instance, it has been used to explore polyphenol metabolism in Solanaceae fruits for breeding purposes [15] and to identify key metabolites in *Lycium barbarum* and *Morchella* [16,17]. While an initial metabolomic study has provided insights into the enzymatic browning of post-harvest *F. velutipes* [18], research on how drying processes regulate the overall metabolism of *F. velutipes* is still lacking. Such knowledge is crucial for directing its processing towards high-value products.

Therefore, this study aimed to systematically investigate the effects of three drying methods (NAD, HAD, and VFD) on the metabolite composition of *F. velutipes* using a metabolomics approach. Concurrently, we evaluated the color change and in vitro antioxidant activity at different drying methods. By integrating these analyses, we sought to delineate the transformation pathways and dynamics of key active components during drying. The findings are expected to provide a scientific basis for elucidating the metabolic mechanisms affected by various drying methods and to support the identification of the most suitable drying technique for maintaining product quality in the food drying industry.

## 2. Materials and Methods

### 2.1. Sample Preparation

#### 2.1.1. Sample Collection

All *F. velutipes* (Mature Fruiting Bodies) purchased in Tianshui Zhongxing Fungi Industry Co., Ltd., (Tianshui, China) the fruiting body of *F. velutipes* was used in this study, and were selected uniformly based on size, color, and mechanical integrity. After cleaning, excess surface moisture was absorbed using absorbent paper, and the samples intended for treatment were separated. The remaining samples were then stored at −80 °C for future use.

#### 2.1.2. Moisture Content Determination

The moisture content was determined based on the method of Nadew [19] with slight modifications: approximately 5 g of the *F. velutipes* sample was weighed and placed in a pre-dried and weighed crucible. The crucible containing the sample was then placed in an oven (H101-3, Shanghai Yuejin Medical Equipment Co., Ltd., Shanghai, China) set at 105 °C ± 2 °C and dried for 4 h to constant weight (Δm < 0.01). After drying, the sample was removed, and its dry weight was measured. The moisture content was calculated using the following formula:

(1) $$\mathrm{MC}\;(\mathrm{wt}\%)\;\frac{W_{0}-W_{d}}{W_{0}}\times 100\;$$

In the formula: MC—moisture content (weight percentage, %); *W*_0_—initial weight of the sample, g; *W*_d_—weight of the sample after drying, g.

#### 2.1.3. Material Drying

CK: Fresh *F. velutipes* were frozen in liquid nitrogen and ground into a fine powder using a pre-chilled mortar and pestle. The homogenate was transferred to a 50 mL centrifuge tube and stored at −80 °C for further analysis. The final moisture content was measured as 89.67% according to Formula (1)

HAD: Fresh *F. velutipes* were evenly placed in an electric blast drying oven (H101-3, Shanghai Yuejin Medical Equipment Co., Ltd., Shanghai, China) and dried at 55 °C for 8 h until a constant weight was achieved. The drying process was considered complete when the weight change between two consecutive measurements, taken 30 min apart, was less than 0.1 g. The final moisture content was measured as 7.56% according to Formula (1).

VFD: Fresh *F. velutipes* were laid flat on the tray without overlapping each other. The fresh *F. velutipes* were pre-cooled at −35 °C for 3 h in a vacuum freeze-drying machine (scientz-20F/A, Ningbo Xinzhi Biotechnology Co., Ltd., Ningbo, China), and then freeze-dried for 45 h under low temperature and vacuum conditions. The final moisture content was measured as 7.62% according to Formula (1).

NAD: Fresh *F. velutipes* were placed flat in a cool, dry place and dried for 36 h to constant weight under controlled conditions (20–25 °C, 28% RH). Drying was considered complete when the weight change between two consecutive measurements was less than 0.1 g. The final moisture content was measured as 7.53% according to Formula (1).

After drying, all samples (HAD, VFD, and NAD) were ground into powder, sieved through a 100-mesh filter, and stored in sealed bags at −20 °C until further analysis.

#### 2.1.4. Extract Preparation

Weighed 1 g of CK, HAD, VFD, and NAD powders accurately. Forty milliliters of 55% anhydrous ethanol was added to each sample. The containers were sealed, and ultrasonic-assisted extraction was performed using an ultrasonic cleaner (SB-5200DTD, Ningbo Xinzhi Biotechnology Co., Ltd., Ningbo, China) at 65 °C for 15 min. After extraction, the mixture was separated by centrifugation at 4500 rpm for 10 min using a VELO18R centrifuge (Lifepower Asia Co., Ltd., Hong Kong, China). The resulting supernatant was transferred to a new centrifuge tube and stored at −20 °C for further analysis, as described in Section 2.3, Section 2.4, Section 2.5 and Section 2.6.

### 2.2. Color Measurement

According to the Galasong method [20], slightly modified, using a CM-700 d colorimeter (Konica Minolta Co., Ltd., Shanghai, China), the instrument was blank proofread before measurement. Each group of samples was measured three times near the equator, and the red-green value color difference a (positive value indicates red bias, negative value indicates green bias), yellow-blue value color difference b (positive value indicates yellow bias, negative value indicates blue bias), and bright value color difference L (the closer to 100, the lighter the color, the closer to 0, the deeper the color) were recorded. The three data points were averaged to obtain the color difference for this group. The saturation C* was calculated according to the values of the color difference a and b. The formula is as follows:

(2) $$C^{*}\;=\;\sqrt{a^{2}+b^{2}}$$

### 2.3. Total Phenol and Total Flavonoids Content Estimation

Total Phenol Content (TPC) was measured using the Folin–Ciocalteu method [21]. The absorbance of gallic acid standard solutions was measured at 760 nm, and a calibration curve was constructed by plotting absorbance against concentration. The curve exhibited good linearity over the range of 0.0 to 0.5 mg/mL, with a regression equation of *y* = 0.6628*x* + 0.07043 and a correlation coefficient of *R*^2^ = 0.998. All experiments were performed in triplicate. The TPC in the sample was expressed as gallic acid equivalents (mg GAE/g dw). The formula is as follows:

(3) $$m=\frac{xnv}{VM}$$

Total Flavonoids Content (TFC) was determined by the NaNO_2_-AlCl_3_-NaOH method [22]. The absorbance of each rutin standard solution was measured at 510 nm, and a calibration curve was constructed by plotting absorbance against concentration. The curve exhibited good linearity over the range of 0.0 to 0.75 mg/mL, with a regression equation of *y* = 0.61421*x* + 0.04742 and a correlation coefficient of *R*^2^ = 0.997. All experiments were performed in triplicate. The determination results of TFC in the sample solution were expressed as rutin equivalent (mg RE/g dw). The calculation formula is the same as Formula (3).

### 2.4. Determination of DPPH Free Radical Scavenging Ability

The scavenging ability of the extract to 2,2-diphenyl-1-trinitrophenylhydrazine (DPPH) free radicals was determined by the method of Odriozola [23]. A 0.1 mM stock solution was obtained by mixing 4 mg DPPH with 100 mL of methanol (95%). The sample (50 mg/mL) and DPPH stock solution were added at a ratio of 1:3, incubated in the dark for 30 min, and the absorbance was recorded at 517 nm. Vitamin C (Vc, 0.125 mg/mL) was used for positive control; the results were expressed as a percentage decrease in absorbance using:

(4) $$R_{DPPH}\left(\%\right)=\frac{A_{1}-A_{2}}{A_{1}}\times 100$$

*A*_1_-blank absorbance, *A*_2_-absorbance of the sample to be tested.

### 2.5. Determination of ABTS Free Radical Scavenging Ability

According to the method of Stunda-Zujeva [24], the ABTS free radical scavenging activity of the extract was determined with Trolox (10–50 Μm dissolved in phosphate buffer, pH 7.4) as the reference compound. ABTS (7 mM) and potassium persulfate (2.45 mM) solutions were prepared with distilled water. The solution was then mixed at a ratio of 1:1 and incubated at room temperature in the dark for 12–16 h. The optical density of the ABTS reagent was adjusted to 0.7~0.74 with distilled water. The sample was added with ABTS at a ratio of 1:3, incubated in the dark for 6 min, and the absorbance was measured at 734 nm. Vc (0.5 mmol/mL) was used for the positive control. The calculation formula is the same as Formula (4).

### 2.6. Determination of Total Antioxidant Capacity

FRAP was analyzed by Xiao [25]. The stock solution was prepared using sodium acetate buffer (300 mM), ferric chloride (20 mM), and 10 mM TPTZ solution (10:1:1). In 96-well plates, 20 µL of four *F. velutipes* extracts and freshly prepared TPTZ solution (280 µL) were added and incubated in darkness at 37 °C for 10 min. The standard curve of ferrous sulfate (FeSO_4_) with a concentration of 15.6 ~ 500 µM was prepared, and the OD value of the sample and the standard was determined at 593 nm. The ΔA standard = standard solution-blank solution was calculated, and the standard curve was made. The results were expressed as extract µmol/mL.

### 2.7. Metabolite Identification and LC-MS Data Analysis

#### 2.7.1. Extraction of LC-MS Samples

100 ± 5 mg of four *F. velutipes* samples were accurately weighed into a 2 mL centrifuge tube, and 800 µL extract (methanol:water = 4:1 (*v*:*v*)) was added for metabolite extraction. The sample solution was ground for 6 min (−10 °C, 50 Hz) on a frozen tissue grinding machine and then extracted using low-temperature ultrasonication for 30 min (5 °C, 40 kHz). The sample was placed at −20 °C for 30 min, centrifuged for 15 min (4 °C, 13,000 *g*), and the supernatant was transferred to a vial with an intubating tube for analysis. In addition, 20 µL of supernatant was removed from each sample and mixed to form a quality control sample.

#### 2.7.2. LC-MS Detection

Using the UHPLC-Q Exactive system (Thermo Fisher Scientific, Waltham, MA, USA), C18 column chromatographic conditions: The chromatographic column was ACQUITY UPLC BEH C18 (100 mm × 2.1 mm i.d., 1.7 µm; Waters, Milford, MA, USA). Mobile phase A was 2% acetonitrile in water (containing 0.1% formic acid), and mobile phase B was acetonitrile (containing 0.1% formic acid). The injection volume was 3 μL, and the column temperature was 40 °C. Sample mass spectrometry signal acquisition was performed using positive and negative ion scanning modes, with a mass scanning range *m*/*z*: 50–1200. The ion spray voltage, the positive ion voltage was 3500 V, the negative ion voltage was −3000 V, the sheath gas was 50 psi, the auxiliary heating gas was 13 psi, the ion source heating temperature was 450 °C, and the 20-40-60 V cycle collision energy was used.

### 2.8. Data Statistics and Analysis

The experimental data were collated and tabulated using Excel 2016, and variance analysis was performed using SPSS (version 24). Graphics were drawn using Origin 2021. All experiments were performed in triplicate. The criterion for a significant difference was *p* < 0.05, and the criterion for an extremely significant difference was *p* < 0.01. LC-MS raw data were preprocessed using Progenesis QI (Waters), and metabolite identification was based on the MJDBPM plant metabolite database for matching. After normalization, differential metabolites were screened using PCA and OPLS-DA analyses, and their potential biological functions were further analyzed using KEGG pathway annotation.

## 3. Results

### 3.1. Effects of Different Drying Methods on the Color of F. velutipes

**Figure 1:**
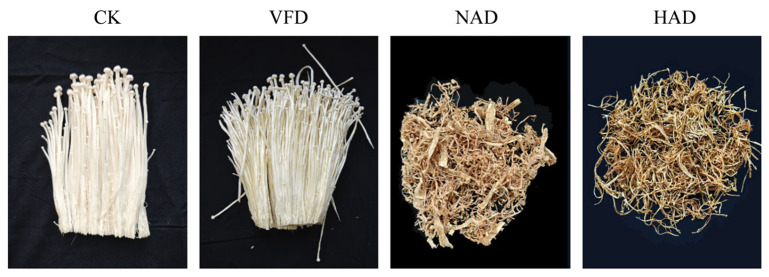
Effects of different drying methods on the color quality of *F. velutipes*.

Enzymatic and non-enzymatic browning are the primary causes of color changes in edible fungi during processing. Enzymatic browning occurs when enzymes, phenolic substrates, and oxygen come into contact, typically following cell damage or at the early stages of storage. In contrast, non-enzymatic browning is mainly induced by heat treatment, involving reactions between sugars and amino acids [26]. In this study, the color of *F. velutipes* changed during the drying process, and the color of *F. velutipes* samples was evaluated. As shown in Figure 1, the overall color of the CK and VFD *F. velutipes* samples was white, the cap was small, the stipe was long, and the color was uniform. The NAD of *F. velutipes* is light brown, and the degree of contraction is relatively small. The HAD *F. velutipes* was yellowish-brown, and the contraction was more obvious Figure 1. At the same time, the color of *F. velutipes* was quantitatively detected using a colorimeter. The color difference parameters of *F. velutipes* under different drying methods are presented in Table 1. The L value of the VFD group was similar to that of the CK group. As the drying temperature increased, both the a value and b value showed an increasing trend, with notable increases observed in the NAD and HAD groups. Regarding color saturation, the C* value was highest in the HAD group and lowest in the VFD group.

### 3.2. Effects of Different Drying Methods on TPC and TFC in F. velutipes

Polyphenols are secondary metabolites produced by plants, including phenolic acids, lignans, coumarins, and flavonoids. Drying leads to changes in total phenols and flavonoids [27]. In this study, the total phenol and flavonoid contents of *F. velutipes* dried using different methods were determined. As shown in Table 2, the TPC and TFC in CK were maintained at low levels, at 17.11 mg GAE/g dw and 3.61 mg RE/g dw, respectively. The drying processes resulted in an increase in the contents of these bioactive compounds. Notably, HAD produced the highest TPC (42.24 mg GAE/g dw), while VFD yielded the highest TFC (7.47 mg RE/g dw). The drying methods differed in their effect on the bioactive compound contents of *F. velutipes*. The overall preservation of total phenols ranked as follows: HAD > VFD > NAD. Similarly, the overall preservation of total flavonoids followed this order: VFD > NAD > HAD.

### 3.3. Effects of Different Drying Methods on Antioxidant Capacity of F. velutipes In Vitro

**Figure 2:**
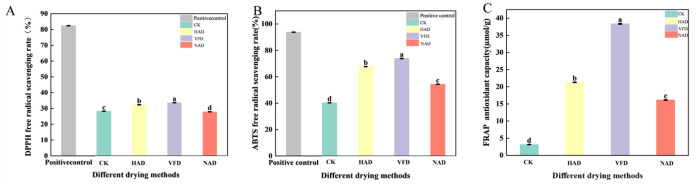
Effects of different drying methods on antioxidant capacity of *F. velutipes* (**A**) DPPH free radical scavenging rate; (**B**) ABTS free radical scavenging rate; (**C**) FRAP antioxidant capacity. Letters indicate significant differences (*p* < 0.001).

Figure 2 shows the evaluation results of the antioxidant capacity of *F. velutipes* under different drying methods. From the overall trend, *F. velutipes* treated by different drying processes exhibited good antioxidant activity, indicating that drying treatment had a positive effect on retaining its functional components. Specifically, the VFD group showed the most prominent performance in DPPH and ABTS free radical scavenging ability and FRAP, with scavenging rates of 33.67%, 73.89%, and 38.35 µmol/g, respectively, which were significantly higher than those of the other groups. The HAD group also demonstrated relatively high antioxidant properties across these assays, slightly lower than the VFD group but still significantly better than the other treatment groups (* *p* < 0.001). Overall, VFD treatment resulted in the best antioxidant capacity.

### 3.4. Effects of Different Drying Methods on Metabolites of F. velutipes

**Figure 3:**
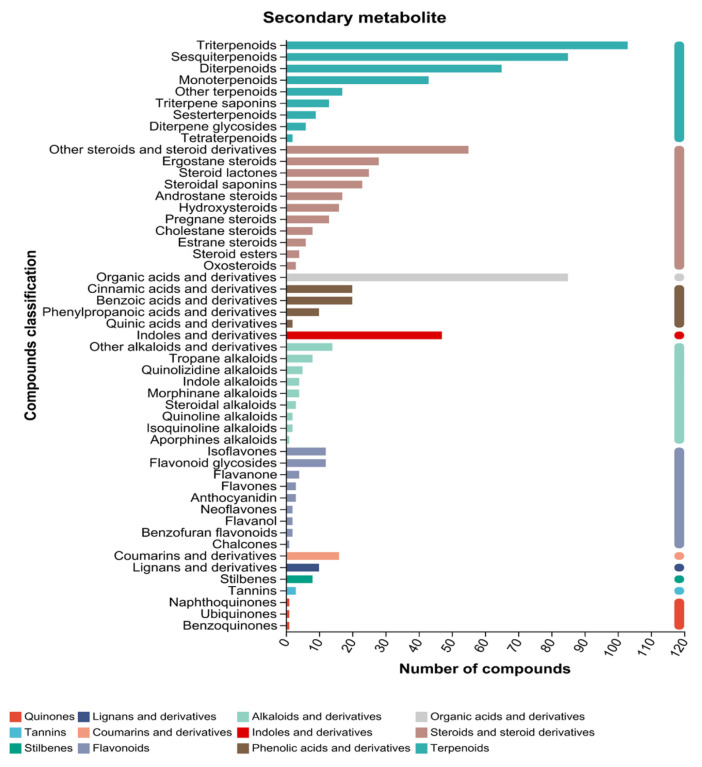
Number of secondary metabolites. The abscissa is the number of metabolites annotated to the species, the ordinate is the secondary classification of plants, and the bar color identifier belongs to the compound primary classification category.

In this study, plant non-targeted metabolomics was used to study the effects of different drying methods on the production and transformation of metabolites in *F. velutipes*. As shown in Table 3, 1430 substances were identified in the positive ion mode, and 1215 substances were identified in the negative ion mode. Lipids (373) and terpenes (343) constituted the main detection population, accounting for 27.1% of the total. The 2645 metabolites were divided into 12 categories of secondary metabolites (Figure 3). These secondary metabolites included 11 steroids, 9 flavonoids, alkaloids and their derivatives, terpenoids, 4 phenolic acids and their derivatives, and 3 quinone compounds.

#### 3.4.1. Principal Component Analysis and Orthogonal Partial Least Squares Discriminant Analysis

**Figure 4:**
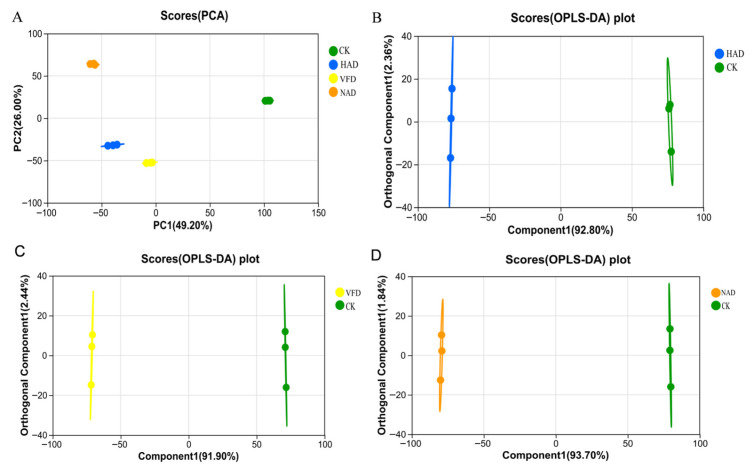
(**A**) PCA; (**B**–**D**) OPLS-DA.

To analyze the overall metabolic differences among samples subjected to different drying methods, Principal Component Analysis (PCA) and Orthogonal Partial Least Squares Discriminant Analysis (OPLS-DA) were applied. PCA provides an overview of metabolic variation among groups and within-group consistency. As shown in Figure 4A, the four groups exhibited clear separation trends, with PC2 explaining 26.00% of the variance, indicating that PC1 played a dominant role in distinguishing the samples. The CK group showed relatively higher scores on both PC1 and PC2, suggesting a more distinct metabolic profile. Samples in the HAD group were more concentrated, indicating good intra-group consistency. The VFD group displayed a distribution pattern similar to that of the HAD group, but was positioned closer to the lower left of the score plot. Meanwhile, the NAD group showed PC1 scores comparable to those of the HAD and VFD groups but had relatively higher PC2 scores, exhibiting a trend closer to that of the CK group.

To further investigate the metabolic differences, supervised OPLS-DA models were constructed to compare HAD vs. CK, VFD vs. CK, and NAD vs. CK (Figure 4B–D). In these models, Comp1 and Orthogonal Comp1 represent the variance explained by the X and Y matrices, respectively. The *Y*-axis variance explained by the orthogonal components was 2.36%, 2.44%, and 1.86%, indicating that most of the intergroup differences were captured along the Comp1 direction. These results demonstrate that different drying methods significantly affected the metabolite profiles of *F. velutipes*, providing a basis for subsequent identification of differential metabolites.

#### 3.4.2. Analysis of Differential Metabolites

**Figure 5:**
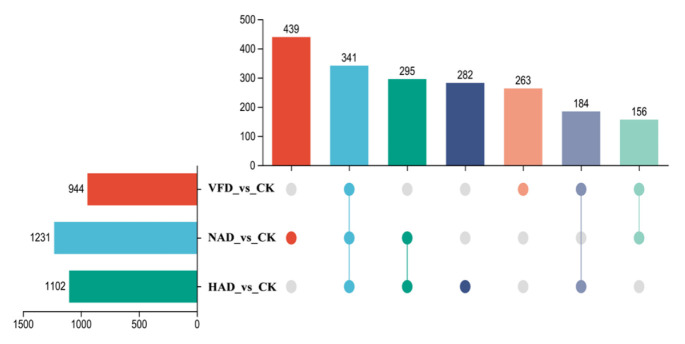
Differential metabolites upset map. The bar chart in the lower-left corner shows the count of elements within each group. The bar chart on the right represents the count of elements resulting from the intersection of different groups. The individual points at the bottom represent elements unique to each group, with lines connecting the points indicating the intersections of unique elements between groups.

To compare metabolite differences among *F. velutipes* samples dried by different methods and identify potential marker metabolites, differential metabolite analysis was performed. As shown in Figure 5, compared with the CK group, VFD, NAD and HAD retained 944, 1231 and 1102 differential metabolites, respectively. Among them, VFD exhibited 263 unique differential metabolites, while NAD and HAD showed 439 and 282 differential metabolites.

**Figure 6:**
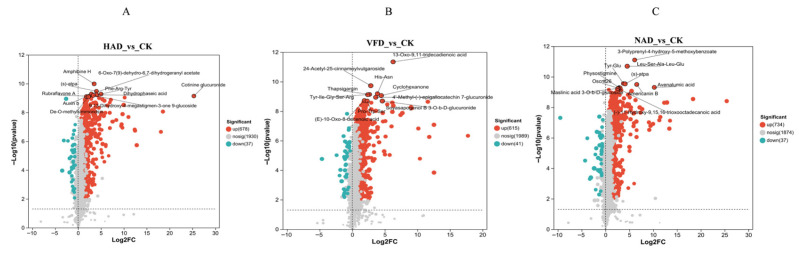
Differential metabolites volcano map (**A**) HAD_vs_CK volcano map; (**B**) VFD_vs_CK volcano map; (**C**) NAD_vs_CK volcano map.

Volcano plot analysis was used to screen significantly differential metabolites that may participate in key metabolic pathways and biological functions. In the HAD group (Figure 6A), 678 metabolites were up-regulated and 37 down-regulated compared to the CK group, with the most notable changes observed in amino acids, peptides, terpenoids, lipids, glycosyl compounds, and flavonoids, including Amphibine H, (S)-atpa and Rubraflavone A. In the VFD group (Figure 6B), 615 metabolites were up-regulated and 41 down-regulated, with significant increases in lipids, terpenoids, and peptides, such as Thapsigargin, 13-oxo-9,11-tridecadienoic acid and Soyasapogenol B 3-O-β-D-glucuronide. The NAD group (Figure 6C) showed 734 up-regulated and 37 down-regulated metabolites, with the most prominent changes in phenolic acids, peptides, lipids, and terpenoids, including 3-polyprenyl-4-hydroxy-5-methoxybenzoate, Leu-Ser-Ala-Leu-Glu and Maslinic acid 3-O-β-D-glucoside. Overall, amino acids, lipids, terpenoids, and peptides were the primary classes of differentially regulated metabolites across all groups.

#### 3.4.3. Metabolite Correlation Analysis

**Figure 7:**
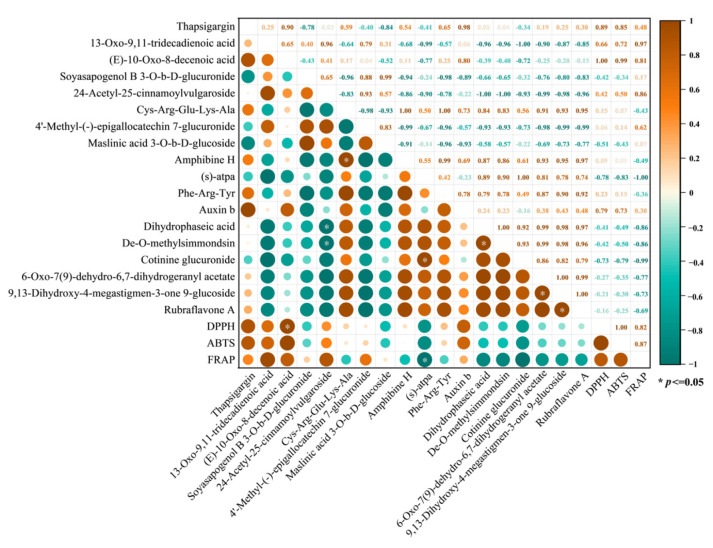
Antioxidant capacity and metabolite correlation diagram (* *p* < 0.05).

Under different drying methods, differentially upregulated metabolites were identified using volcano plots, and their relationships with the antioxidant capacity of *F. velutipes* were further evaluated through correlation analysis (Figure 7). Correlation analysis showed that three antioxidant indicators, DPPH radical scavenging activity, ABTS radical scavenging activity, and FRAP, were significantly and positively correlated with Thapsigargin (organic acids), (E)-10-oxo-8-decenoic acid, 13-oxo-9,11-tridecadienoic acid (lipids), and 24-Acetyl-25-cinnamoylvulgaroside (terpenoids). In contrast, these antioxidant indicators were significantly negatively correlated with (S)-atpa (amino acids), Dihydrophaseic acid (terpenoids), De-O-methylsimmondsin, and Cotinine glucuronide (carbohydrates and derivatives).

#### 3.4.4. Cluster Heat Map Analysis of Differential Metabolites

**Figure 8:**
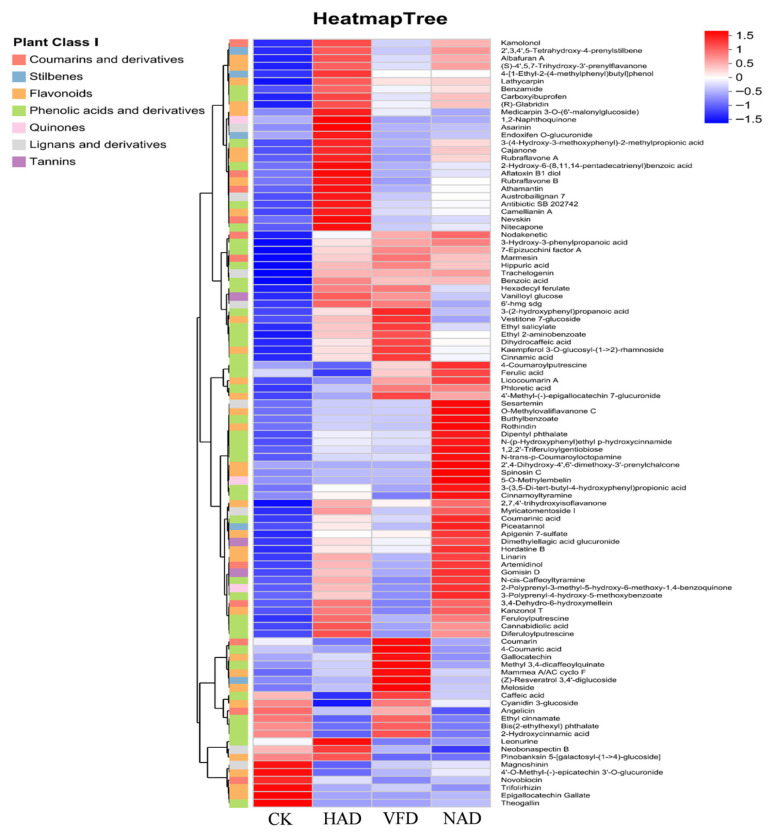
Cluster heat map of phenolic differential metabolites of *F. velutipes* under different drying methods.

In order to more specifically understand the differences in metabolites of *F. velutipes* under different drying methods, this study performed cluster heat map analysis on 11 categories of lipids, terpenoids, amino acids, organic acids, sterols, phenolic acids, and their derivatives with different significance. As shown in Figure S1, metabolomics analysis revealed that different drying methods have profoundly reshaped the metabolic composition of *F. velutipes*. The metabolic characteristics of CK were dominated by structural and primary metabolic substances, including membrane lipid components (Glycerol 2-phosphate, LysoPE) and free amino acids (L-norleucine, L-glutathione). These metabolites are closely associated with the maintenance of cellular membrane integrity and the regulation of key metabolic pathways required for growth, reproduction, and cellular defense, reflecting an active primary metabolic state in CK [28,29]. After drying, the enriched metabolites shifted towards bioactive-related metabolites, mainly including polyphenols, terpenoids, and organic acids. Notably, different drying methods led to changes in the accumulation patterns of secondary metabolites. Analysis of phenolic metabolites revealed distinct accumulation patterns under different drying methods (Figure 8). In the HAD group, specific phenolic compounds were significantly upregulated, including the lignan asarinin and several flavonoid glycosides. Conversely, simple phenolic acids such as caffeic acid were not enriched in HAD samples. The VFD group exhibited a diverse and complete profile of phenolic acids, including caffeic acid, 4-coumaric acid, and their esters, along with the heat-sensitive anthocyanin cyanidin 3-glucoside. Coumarin was also enriched in VFD-treated samples. In the NAD group, unique isoflavones (e.g., O-methylovali flavanone C) and phenolic esters (e.g., 1,2,2′-triferuloylgentiobiose) accumulated.

#### 3.4.5. KEGG Pathway Analysis

**Figure 9:**
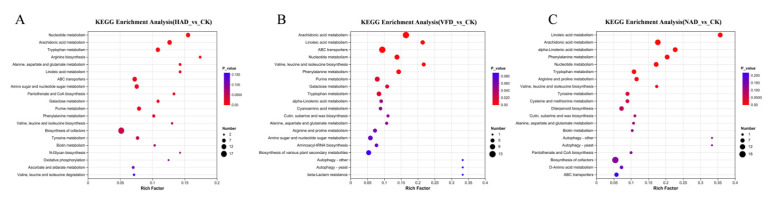
KEGG enrichment pathway (**A**) HAD_vs_CK KEGG enrichment analysis; (**B**) VFD_vs_CK KEGG enrichment analysis; (**C**) NAD_vs_CK KEGG enrichment analysis.

KEGG pathway enrichment analysis, from the metabolic perspective, provides an intrinsic mechanism explanation and a systematic background for the observed accumulation patterns of metabolites. In the enrichment analysis, the *x*-axis represents the enrichment rate, while the *y*-axis shows the KEGG pathways. The size of each bubble in the figure indicates the number of compounds enriched in the metabolic concentration within the pathway. The color of the bubble reflects the *p*-value of the enrichment significance; a smaller *p*-value corresponds to higher enrichment significance. As illustrated in Figure 9, compared to the CK group, the VFD group was primarily enriched in pathways related to Arachidonic acid metabolism, Linoleic acid metabolism, ABC transporter metabolism, Nucleotide metabolism, and Phenylalanine metabolism (Figure 9B). The NAD group showed enrichment mainly in Linoleic acid metabolism, Arachidonic acid metabolism, α-Linolenic acid metabolism, Phenylalanine metabolism, and Nucleotide metabolism pathways (Figure 9C). The HAD group was predominantly enriched in Nucleotide metabolism, Arachidonic acid metabolism, Tryptophan metabolism, Arginine biosynthesis, and the metabolic pathways of Alanine, aspartic acid, and glutamic acid (Figure 9A).

#### 3.4.6. Analysis of Key Metabolites

**Figure 10:**
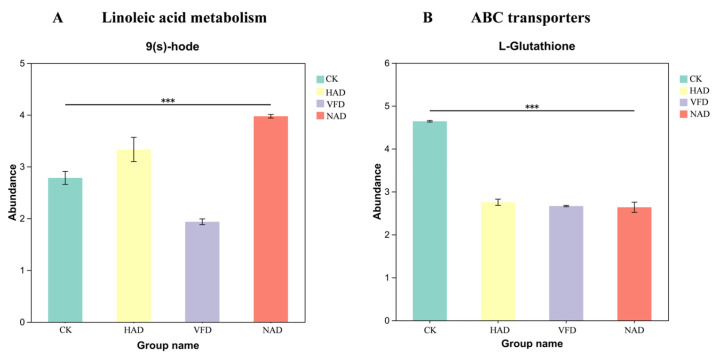
Relative abundance of key metabolites in different drying methods: (**A**) the content of Linoleic acid metabolism in CK, HAD, VFD, and NAD; (**B**) the content of ABC transporters in CK, HAD, VFD, and NAD. (*** *p* < 0.001).

Based on the KEGG enrichment results, this study found critical differential metabolites within pathways, offering insights into the potential metabolic shifts influenced by the drying methods. The abundance of representative metabolites from nucleotide metabolism, phenylalanine metabolism, linoleic acid metabolism, and α-linolenic acid metabolism exhibited distinct variation patterns across the CK, HAD, VFD, and NAD groups (Figure S2). In the Linoleic acid metabolism (Figure 10A), 9(s)-HODE levels increased markedly in the HAD and NAD groups, while remaining relatively unchanged in the VFD group. In the ABC transporter pathway, L-Glutathione was identified as a key differential metabolite across all drying treatments. As shown in Figure 10B, the relative content of L-Glutathione remained consistent in the CK, HAD, VFD, and NAD groups, indicating that this metabolite was not significantly affected by the drying methods.

## 4. Discussion

Different drying methods can lead to color changes, which are related to the drying temperature and the content of active ingredients [30]. The results showed that the three drying methods had significant effects on the color and shrinkage of *F. velutipes*, which indicated that VFD better preserves the appearance, shape, and color of fresh *F. velutipes* before drying. At the same time, VFD samples showed superior resistance to color degradation, which was due to the low temperature and vacuum environment, which inhibited the degradation or browning reaction of heat-sensitive pigments [31]. During the drying process, NAD and HAD were exposed to oxygen for extended periods at high temperatures, leading to browning reactions and pigment degradation, which caused significant changes in the color and morphology of *F. velutipes*. The similarity in L values between the VFD and CK groups indicates that vacuum freeze drying effectively preserves the original brightness of the samples. This suggests that the VFD process helps maintain color stability by minimizing pigment degradation or browning reactions. Additionally, the observed increases in a and b values with rising drying temperatures, particularly in the NAD and HAD groups, indicate that thermal drying processes induce color changes due to non-enzymatic browning or pigment concentration. This difference can be attributed to heat-sensitive substances. Literature [32] indicates that the color changes observed during drying are mainly attributed to non-enzymatic browning caused by the Maillard reaction at elevated temperatures.

Statistical analysis revealed that the drying method significantly influenced the increase in bioactive compounds. In the early stages of HAD, enzymatic oxidation and thermal degradation may occur; however, high temperatures activate the biosynthetic pathways of phenolic compounds (such as the phenylpropanoid metabolism pathway), with the synthesis rate surpassing the degradation rate, resulting in a net accumulation [33]. Flavonoids are prone to degradation under high-temperature conditions due to their low thermal stability, leading to a significant decrease in their content. This also explains why TFC is reduced in HAD [34]. In contrast, the VFD method causes less damage to the organizational structure of the *F. velutipes*. The preservation of a more intact cellular structure in VFD helps maintain the overall appearance of the product. However, the more complete structure in the final product may hinder the release of phenolic compounds during extraction. This structural barrier limits the availability of bioactive compounds, resulting in a lower TPC despite the intact appearance [35]. For NAD, the prolonged drying time under ambient conditions allows for gradual biosynthesis of phenolic compounds, but the sustained exposure to oxygen may also lead to oxidative degradation over time, resulting in the lowest increase among the three methods. These results suggest the following guidelines for selecting drying methods: prioritize the VFD method to preserve flavonoids, or use the HAD method to enhance the transformation and enrichment of phenolic compounds in *F. velutipes*.

To further explain the observed differences in antioxidant capacity among the drying treatments, the relevant literature was reviewed. In the present study, the excellent antioxidant performance of VFD can be attributed to the protective effect of low-temperature drying on heat-sensitive bioactive compounds. Phenolic compounds and total flavonoids, known for their strong antioxidant properties, are thermolabile and prone to degradation under high temperatures. The relatively low temperature of the VFD process effectively minimizes the thermal degradation of these compounds, thereby enhancing their retention and antioxidant efficacy [36]. In line with this, our metabolomic analysis revealed that VFD treatment preserved a more complete profile of phenolic acids and heat-sensitive flavonoids (Figure 5), both of which are key contributors to antioxidant capacity. Additionally, Shahin et al. [27] emphasized that high-temperature and prolonged drying not only inactivates enzymes but also induces the non-enzymatic degradation of several heat-sensitive compounds. This finding aligns with our observations that, although HAD and NAD treatments led to some accumulation of phenolic polymers, their overall antioxidant activity was significantly lower than that of the VFD samples. This is likely due to the thermal degradation of labile antioxidant components during the higher-temperature drying processes.

In this study, untargeted metabolomics was used to examine the effects of different drying methods on the metabolite profiles of *F. velutipes*. Lipids are not only major structural components of cellular membranes but are also closely associated with the formation of flavor compounds during postharvest processing [37]. Terpenoid compounds, on the other hand, are widely recognized for their diverse biological activities, including antioxidant and antimicrobial properties [38]. The relatively high abundance of these metabolites suggests that they may play important roles in shaping the quality characteristics of *F. velutipes* during the drying process. In addition, several classes of bioactive secondary metabolites were detected, including flavonoids, alkaloids, phenolic acids, and quinones. These compounds are generally associated with the antioxidant capacity and health-promoting properties of *F. velutipes* [39,40]. Previous studies have indicated that drying temperature can significantly influence the stability and transformation of metabolites [41]. Therefore, the presence of these secondary metabolites in the present study suggests that different drying methods may affect their accumulation and conversion, ultimately influencing the nutritional value and functional properties of the dried products. PCA and OPLS-DA analyses revealed significant differences in metabolite profiles among the CK, HAD, VFD, and NAD groups, suggesting that different drying methods substantially influenced the metabolic composition of *F. velutipes*.

In the analysis of differential metabolites, the metabolite profiles of *F. velutipes* samples subjected to different drying methods were compared. The results indicated that each drying method preserved a distinct set of metabolites. Volcano plot analysis highlighted key metabolites involved in important metabolic pathways. Overall, amino acids, lipids, terpenoids, and peptides emerged as the primary classes of differentially regulated metabolites.

The observed correlations suggest that specific metabolites generated under different drying methods may contribute differently to the antioxidant potential of *F. velutipes*. The positive correlations observed indicate that these metabolites likely play a significant role in enhancing the mushroom’s antioxidant capacity. Lipids and lipid-derived metabolites are commonly involved in oxidative stress responses and redox signaling in biological systems, while terpenoid compounds have been widely reported to exhibit antioxidant activities in natural products [42,43]. Therefore, the enrichment of these metabolites during certain drying processes may partially account for the enhanced antioxidant activity observed in dried samples. Overall, the results indicate that drying-induced metabolic changes strongly influence the antioxidant characteristics of *F. velutipes*.

The clustering results revealed distinct grouping patterns. The significant upregulation of lignans and specific flavonoid glycosides in the HAD group might be attributed to the activation of the phenylpropanoid metabolic pathway by high temperature and subsequent glycosylation/polymerization reactions [44]. These newly formed phenolic polymers might contribute to the typical dark color of HAD samples along with Maillard reaction products. The lack of enrichment of simple phenolic acids (such as caffeic acid) in HAD is possibly due to their degradation or transformation at high temperatures. The enrichment of a rich and complete phenolic acid profile, as well as heat-sensitive anthocyanins, in the VFD group is likely due to the low-temperature vacuum environment, which greatly inhibits enzymatic oxidation and thermal degradation, allowing VFD products to retain the natural antioxidant potential and lighter color of fresh *F. velutipes* to the greatest extent. The enrichment of coumarin in VFD might also contribute to its unique flavor. This finding is consistent with a previous study reporting that VFD samples exhibited a wider variety of metabolites. The VFD process, carried out under low temperature and vacuum conditions, effectively avoids the degradation and transformation of heat-sensitive compounds caused by high temperature, while significantly inhibiting oxidation reactions and protecting easily oxidized metabolites. This view is further supported by a study on *Pleurotus citrinopileatus*, which found that VFD-treated samples retained the most complete metabolites, including umami amino acids, antioxidant phenols, and nucleotides [45]. The accumulation of unique isoflavones and phenolic esters in the NAD group may result from slow oxidation reactions occurring during the mild and prolonged drying process. This process might endow NAD with more complex and rich flavor precursors, but it might also lead to the loss of some active substances due to slow oxidation. The clustering patterns observed in the heatmap indicate that the drying method determines the extent of thermal degradation, enzymatic browning, and oxidative stress, thereby selectively shaping the accumulation or depletion of specific metabolite categories.

KEGG metabolic pathway analysis further revealed that the drying temperature significantly influenced the metabolic pathways of *F. velutipes*. Notably, the distinct patterns of pathway enrichment induced by different drying methods were consistent with previous findings [45]. There are significant differences in the number and types of metabolic pathways; this directly proves that the drying method is a key factor in regulating the differences in metabolic pathways of *F. velutipes*. The distinct KEGG pathway enrichment profiles observed across the VFD, NAD, and HAD groups indicate that the drying method not only influences individual metabolite abundances but also systematically reprograms global metabolic networks. Specifically, the shift from the dominance of primary metabolism in HAD samples to the preservation of secondary metabolism in VFD samples underscores the profound, pathway-specific impact of processing conditions on the nutritional and functional properties of the final product.

In addition, this study also found that the temperature difference between different drying methods is a key factor in regulating the core metabolic nodes, which further affect the accumulation of upstream or downstream important metabolites by regulating the expression of key enzymes. Taking L-Glutathione in the ABC transporter pathway as an example, it was significantly down-regulated in all drying treatments, indicating that temperature was the main cause of its depletion. This result is consistent with the report of Lin et al. that L-Glutathione is easily degraded at room temperature, and the oxidation reaction still occurs at −20 °C, which further supports the effect of temperature on the stability of the metabolite [46]. On the other hand, in the linoleic acid metabolic pathway, the content of 9(s)-HODE decreased under VFD conditions, while it increased significantly in HAD and NAD. It is established that the formation of 9(s)-HODE depends on the catalytic activity of the ALOX15 enzyme [47]. In this study, VFD was carried out at an ultra-low temperature of −35 °C, which significantly inhibited the enzyme activity of ALOX15 and led to a decrease in 9(s)-HODE synthesis. On the contrary, both HAD and NAD are in the appropriate active temperature range of ALOX15, which can effectively catalyze linoleic acid to 9(s)-HODE. In addition, the samples were exposed to air during the HAD and NAD processes, which further promoted the enzymatic reaction and eventually led to an increase in 9-HODE content.

In summary, VFD demonstrated superior performance in preserving the attributes of *F. velutipes*, including color, appearance, antioxidant activity and rich metabolite composition, although it requires a much longer processing time (45 h). While HAD (8 h) and NAD (36 h) offer advantages in processing efficiency and lower operational costs, they are associated with greater quality degradation. Nevertheless, the high equipment investment and longer processing time required for VFD may limit its large-scale industrial application. Therefore, both product quality and economic feasibility should be considered when selecting an appropriate drying method for *F. velutipes*. Therefore, this study focused on characterizing the final dried products, without monitoring the drying process in real time. However, drying rate plays a key role in the degradation and transformation of secondary metabolites, as it affects temperature, water activity, and oxygen exposure. Differences in metabolite profiles between drying methods likely result from the combined effects of drying temperature, time, and rate. Future work should track drying kinetics and link drying rate to metabolite changes, which would help clarify how key quality components form and transform, and support more precise control of the drying process.

## 5. Conclusions

This study demonstrates that different drying methods have significant impacts on the color, bioactive compound content, antioxidant capacity, and metabolites of *F. velutipes*. VFD effectively preserves the *F. velutipes*’ appearance, shape, and color, while minimizing the degradation of heat-sensitive pigments, resulting in superior color stability. VFD also maintains higher levels of phenolic compounds and flavonoids, key antioxidants, by protecting them from thermal degradation and enzymatic oxidation. In contrast, HAD and NAD can promote the accumulation of certain metabolites, particularly phenolic polymers, but are associated with noticeable color changes and reduced antioxidant activity due to high-temperature exposure and prolonged contact with oxygen. These findings highlight the importance of selecting drying methods based on the desired quality attributes. VFD is the most suitable method for preserving the nutritional and functional properties of *F. velutipes,* including its antioxidant capacity and bioactive compounds, whereas HAD may be preferred when the goal is to promote the transformation and enrichment of phenolic compounds. This study provides a scientific foundation for selecting appropriate postharvest drying methods for *F. velutipes*.

## Figures and Tables

**Table 1:** Color parameters of *F. velutipes* under different drying methods.

Different Drying Methods	L	a	b	C*
CK	78.10 ± 0.08 ^B^	−1.53 ± 0.01 ^D^	9.8 ± 0.04 ^D^	9.92 ± 0.04 ^D^
HAD	43.18 ± 0.09 ^D^	12.15 ± 0.04 ^A^	21.73 ± 0.04 ^A^	24.90 ± 0.03 ^A^
VFD	89.73 ± 0.02 ^A^	−0.31 ± 0.02 ^C^	15.98 ± 0.05 ^C^	15.98 ± 0.05 ^C^
NAD	46.70 ± 0.03 ^C^	10.25 ± 0.04 ^B^	19.23 ± 0.06 ^B^	21.78 ± 0.06 ^B^

Note: ^ABCD^ in the table indicates significance.

**Table 2:** Effects of different drying treatments on TPC and TFC of *F. velutipes.*

**Different Drying Methods**	**TPC (mg GAE/g dw)**	**TFC (mg RE/g dw)**
CK	17.11 ± 0.0003 ^D^	3.61 ± 0.0004 ^D^
HAD	42.24 ± 0.0012 ^A^	5.06 ± 0.0003 ^C^
VFD	37.50 ± 0.0017 ^B^	7.47 ± 0.0002 ^A^
NAD	31.22 ± 0.0011 ^C^	5.32 ± 0.0003 ^B^

Note: ^ABCD^ in the table indicates significance.

**Table 3:** Classification of all metabolites identified in this study.

Classification of Metabolites	Negative Ion Mode	Positive Ion Mode	Total
Lipids	181	192	373
Terpenoids	128	215	343
Amino acids and derivatives	99	104	203
Carbohydrates and derivatives	83	47	130
Steroids and steroid derivatives	79	119	198
Organic acids and derivatives	55	30	85
Nucleotides and derivatives	34	33	67
Phenolic acids and derivatives	29	23	52
Flavonoids	29	12	41
Indoles and derivatives	25	22	47
Alkaloids and derivatives	17	26	43
Coumarins and derivatives	8	8	16
Lignans and derivatives	6	4	10
Vitamins	4	7	11
Stilbenes	4	4	8
Quinones	3	0	3
Tannins	2	1	3
Others	429	583	1012
Total	1215	1430	2645

## Data Availability

The original contributions presented in the study are included in the article/Supplementary Materials, further inquiries can be directed to the corresponding author.

## Supplementary Materials

The following supporting information can be downloaded at https://www.mdpi.com/article/10.3390/foods15071208/s1. Figure S1: Cluster heat map of differential metabolites of *F. velutipes* under different drying methods; Figure S2: Relative abundance of key metabolites in different drying methods.

## Abbreviations

The following abbreviations are used in this manuscript:

CK	Fresh *F. velutipes* the control group
HAD	Hot-Air-Dried
VFD	Vacuum-Freeze-Dried
NAD	Natural-Air-Dried
TPC	Total Phenol Content
TFC	Total Flavonoids Content
PCA	Principal Component Analysis
OPLS-DA	Orthogonal Partial Least Squares Discriminant Analysis
