# Supplementary material for: Effects of Various Drying Techniques on the Quality Attributes and Metabolite Profiles of Flammulina velutipes (Fruiting Body) Based on Non-Target Metabolomics
Source: Foods. 2026 Apr 2;15(7):1208. doi: 10.3390/foods15071208 (PMC13073847; doi:10.3390/foods15071208)

Figure S1. Cluster heat map of differential metabolites of *F.velutipes* under different drying methods

Figure S2. Relative abundance of key metabolit es in different drying methods

Figure S1. Cluster heat map of differential metabolites of *F.velutipes* under different drying methods

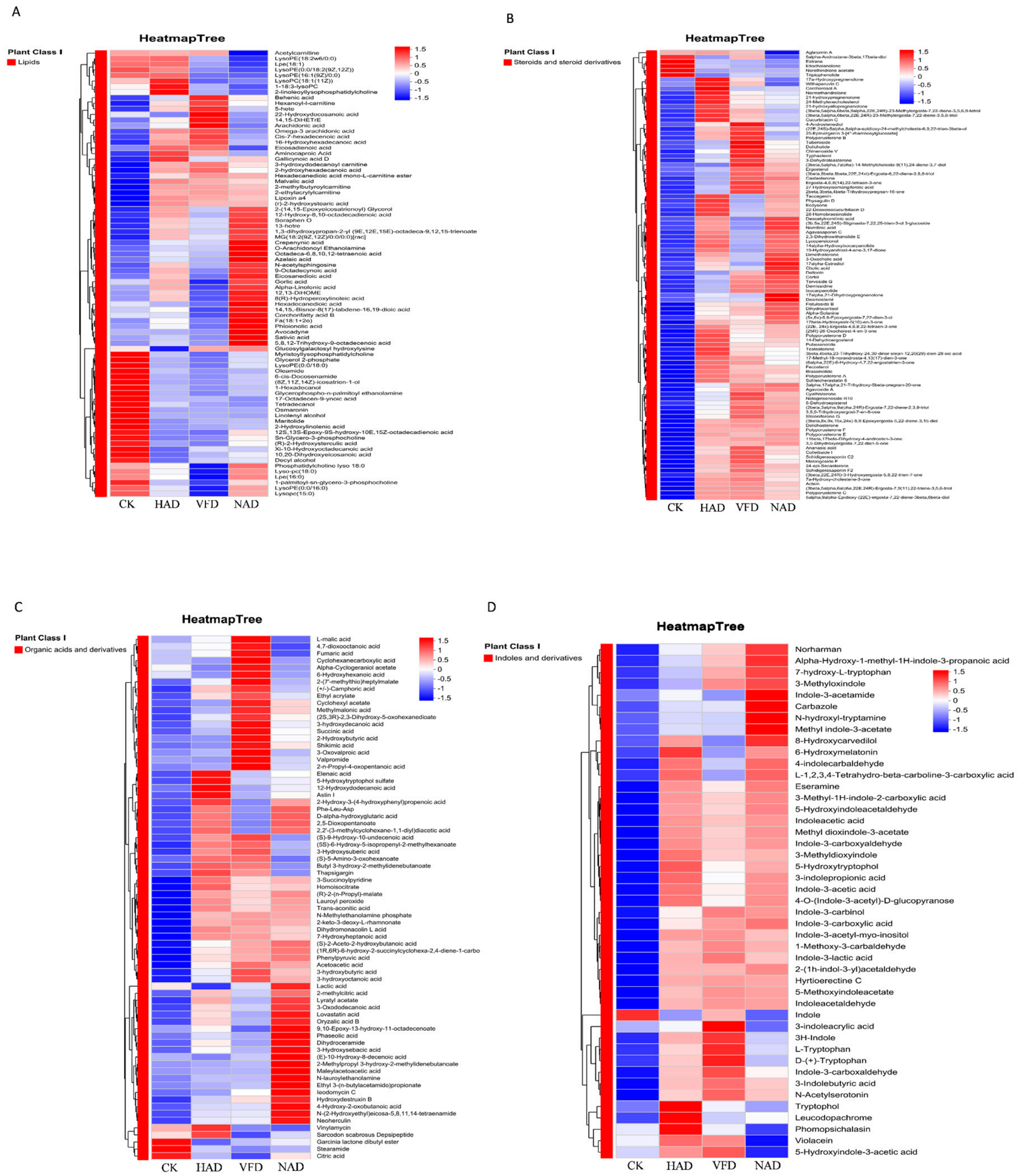

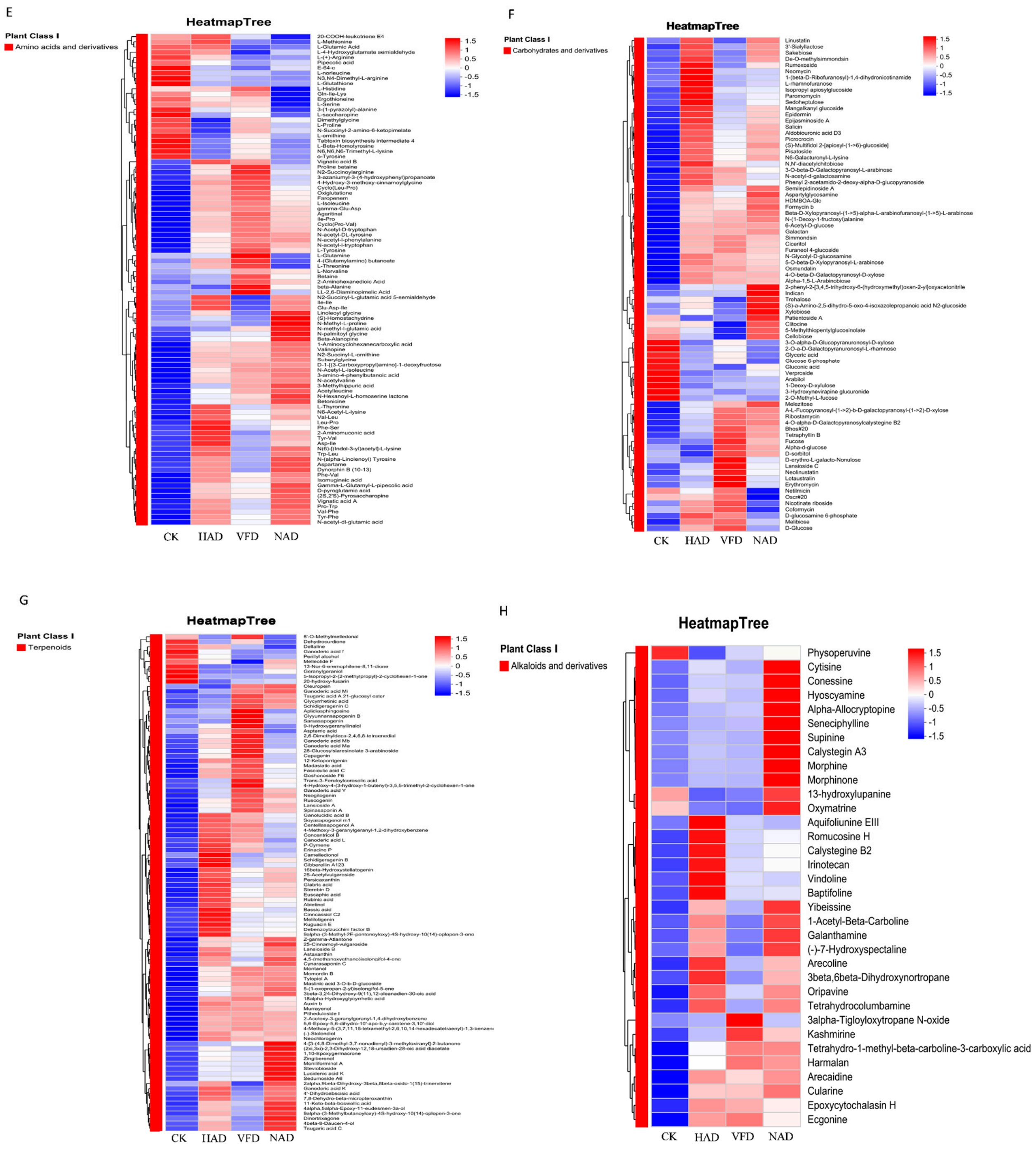

E
HeatmapTree
Plant Class I
Amino acids and derivatives
1.5
1
0.5
0
-0.5
-1
-1.5
CK
HAD
VFD
NAD
F
HeatmapTree
Plant Class I
Carbohydrates and derivatives
1.5
1
0.5
0
-0.5
-1
-1.5
CK
HAD
VFD
NAD
G
HeatmapTree
Plant Class I
Terpenoids
1.5
1
0.5
0
-0.5
-1
-1.5
CK
HAD
VFD
NAD
H
HeatmapTree
Plant Class I
Alkaloids and derivatives
Physoperuvine
Cytisine
Conessine
Hyoscyamine
Alpha-Allocryptopine
Senecіphylline
Supinine
Calystegin A3
Morphine
Morphinone
13-hydroxylupanine
Oxymatrine
Aquifoliunine EIII
Romucosine H
Calystegine B2
Irinotecan
Vindoline
Baptifoline
Yibeissine
1-Acetyl-Beta-Carboline
Galanthamine
(-)-7-Hydroxyspectaline
Arecoline
3beta,6beta-Dihydroxynortropane
Oripavine
Tetrahydrocolumbamine
3alpha-Tigloyloxytropane N-oxide
Kashmirine
Tetrahydro-1-methyl-beta-carboline-3-carboxylic acid
Harmalan
Arecaidine
Cularine
Epoxycytochalasin H
Ecgonine
1.5
1
0.5
0
-0.5
-1
-1.5
CK
HAD
VFD
NAD

I

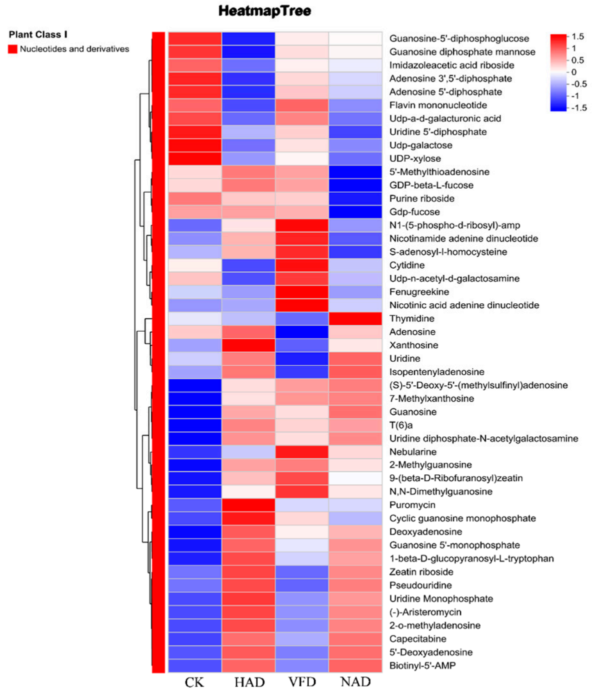
HeatmapTree
Plant Class I
Nucleotides and derivatives
1.5
1
0.5
0
-0.5
-1
-1.5
Guanosine-5'-diphosphoglucose
Guanosine diphosphate mannose
Imidazoleacetic acid riboside
Adenosine 3',5'-diphosphate
Adenosine 5'-diphosphate
Flavin mononucleotide
Udp-a-d-galacturonic acid
Uridine 5'-diphosphate
Udp-galactose
UDP-xylose
5'-Methylthioadenosine
GDP-beta-L-fucose
Purine riboside
Gdp-fucose
N1-(5-phospho-d-ribosyl)-amp
Nicotinamide adenine dinucleotide
S-adenosyl-l-homocysteine
Cytidine
Udp-n-acetyl-d-galactosamine
Fenugreekine
Nicotinic acid adenine dinucleotide
Thymidine
Adenosine
Xanthosine
Uridine
Isopentenyladenosine
(S)-5'-Deoxy-5'-(methylsulfinyl)adenosine
7-Methylxanthosine
Guanosine
T(6)a
Uridine diphosphate-N-acetylgalactosamine
Nebularine
2-Methylguanosine
9-(beta-D-Ribofuranosyl)zeatin
N,N-Dimethylguanosine
Puromycin
Cyclic guanosine monophosphate
Deoxyadenosine
Guanosine 5'-monophosphate
1-beta-D-glucopyranosyl-L-tryptophan
Zeatin riboside
Pseudouridine
Uridine Monophosphate
(-)-Aristeromycin
2-o-methyladenosine
Capecitabine
5'-Deoxyadenosine
Biotinyl-5'-AMP
CK
HAD
VFD
NAD

J

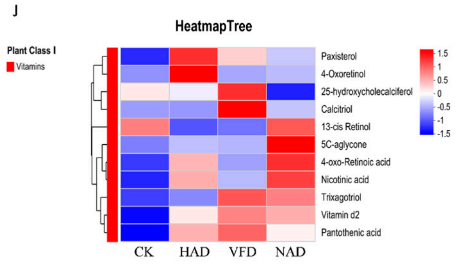
HeatmapTree
Plant Class I
Vitamins
1.5
1
0.5
0
-0.5
-1
-1.5
Paxisterol
4-Oxoretinol
25-hydroxycholecalciferol
Calcitriol
13-cis Retinol
5C-aglycone
4-oxo-Retinoic acid
Nicotinic acid
Trixagotriol
Vitamin d2
Pantothenic acid
CK
HAD
VFD
NAD

Figure S2. Relative abundance of key metabolit es in different drying methods

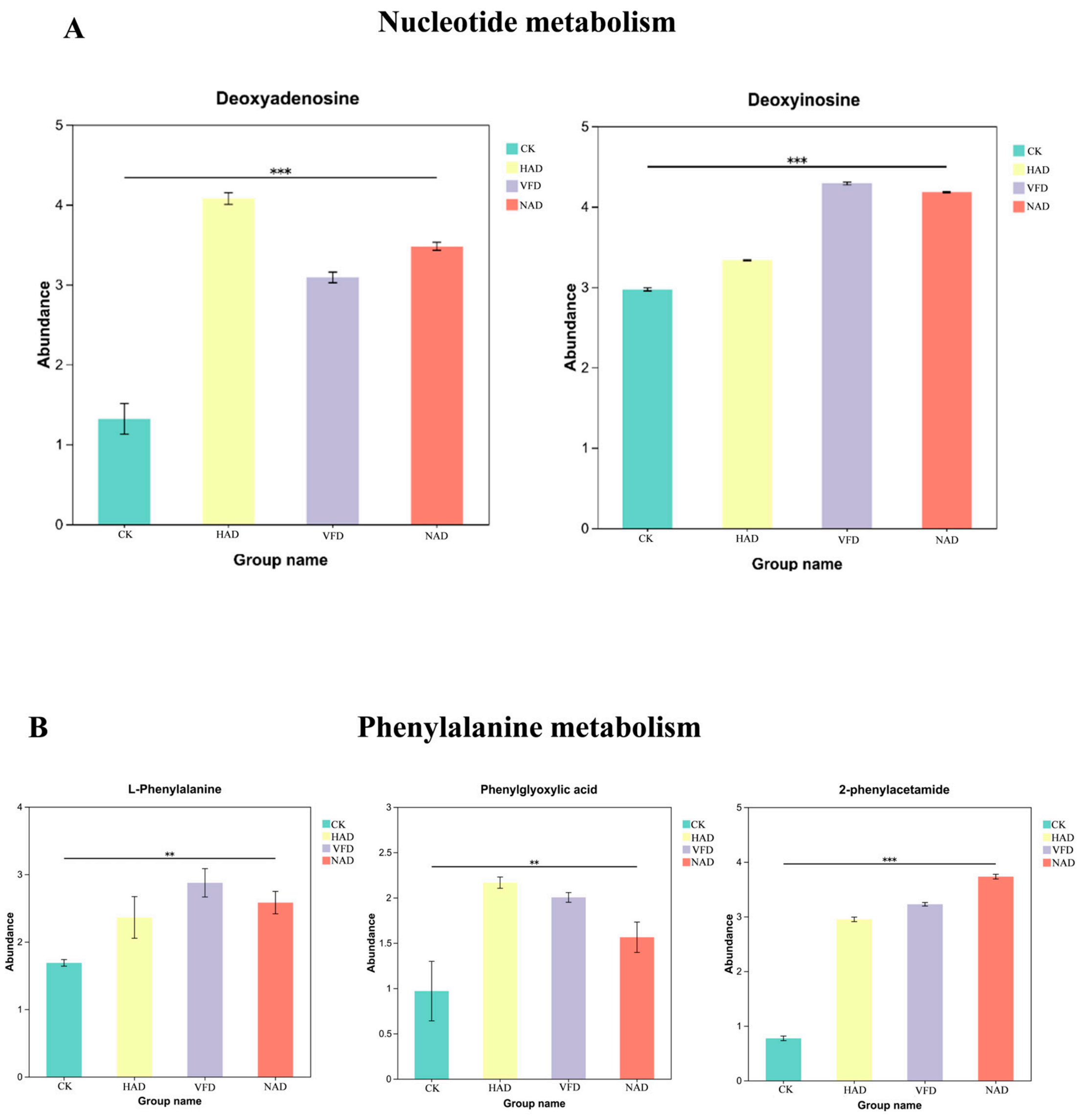

C Linoleic acid metabolism

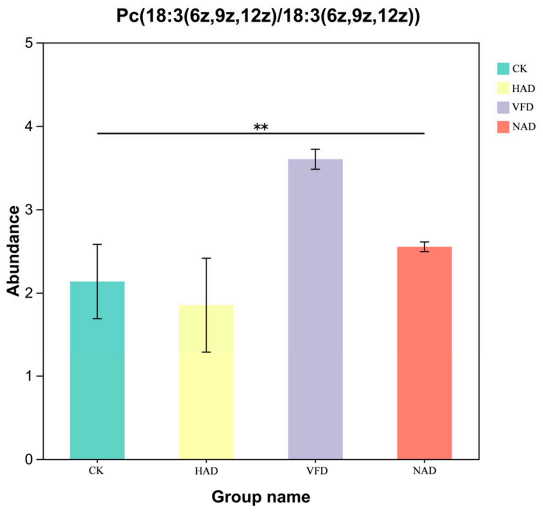

D alpha-Linolenic acid metabolism

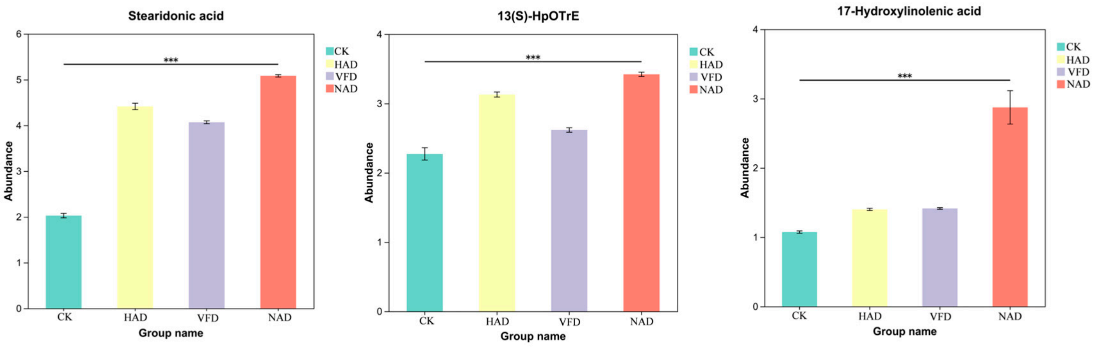

E

Arachidonic acid metabolism

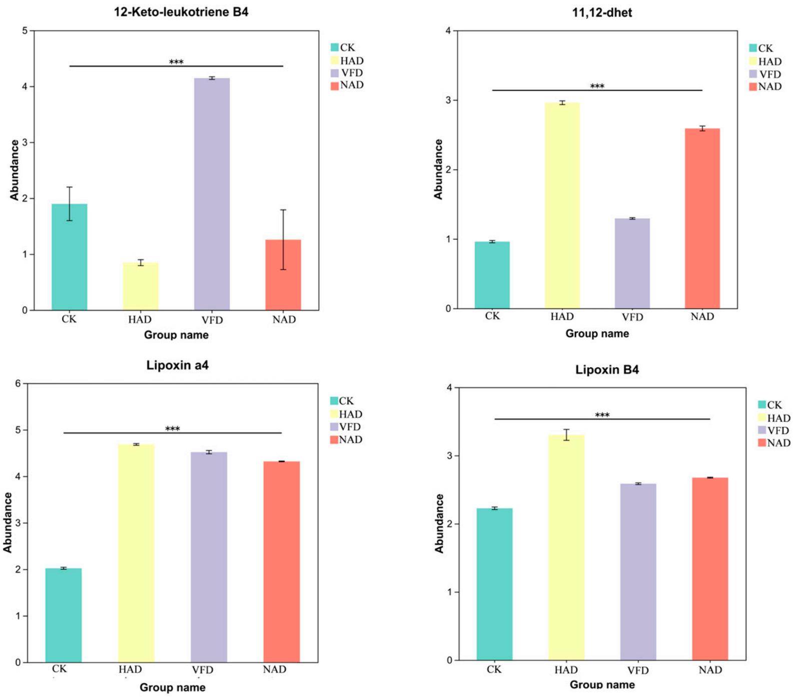

Supplement: Supplementary file 1 [file foods-15-01208-s001.zip › foods-4170922-supplementary.pdf]
